# A digital ELISA for multiplexed detection of allergen‐specific IgE against Der p 1, Der p 2, and Der p 23

**DOI:** 10.1002/btm2.70068

**Published:** 2025-08-29

**Authors:** Feifei Han, Shih‐Mo Yang, Ju Xue, Wanying Xie, Yuanfen Liao, Qi Cheng, Dongmei Zhou, Chuanlu Ren, Yubao Cui

**Affiliations:** ^1^ Department of Clinical Laboratory The Affiliated Wuxi People's Hospital of Nanjing Medical University Wuxi China; ^2^ School of Mechatronic Engineering and Automation Shanghai University Shanghai China; ^3^ Department of Laboratory The 904th Hospital of the Joint Logistics Support Force of Chinese People's Liberation Army Wuxi China; ^4^ Clinical Research Center The Affiliated Wuxi People's Hospital of Nanjing Medical University Wuxi China

**Keywords:** component‐resolved diagnosis, *Dermatophagoides pteronyssinus*, digital ELISA, house dust mite (HDM), microfluidic chip

## Abstract

The house dust mite *Dermatophagoides pteronyssinus* produces major allergens (Der p 1, Der p 2, and Der p 23) that require precise IgE detection for clinical diagnosis. We developed a multiplex digital ELISA using fluorescence‐encoded micromagnetic beads (532 nm/638 nm dual‐wavelength system) coupled with microfluidics to simultaneously quantify serum IgE against these components, with comprehensive evaluation against the clinical standard UniCAP system. The 532 nm channel measured allergen‐specific signals via average brightness increase (ABMB) of enzymatically amplified fluorescence, while 638 nm enabled spectral bead differentiation. Comparative evaluation with UniCAP showed the improved digital ELISA achieved uniform 75.0% sensitivity but variable specificity (42.9%–54.5%) across allergens at the 15.8% ABMB threshold. Sample classification results (Der p 1: 9 positive/6 negative; Der p 2: 7/8; Der p 23: 7/8) demonstrated suboptimal positive predictive values (33.3%–60.0%) versus more favorable negative predictive values (60.0%–85.7%), with likelihood ratios (LR+: 1.31–1.65) and Cohen's *κ* (0.12–0.25) suggesting limited diagnostic reliability. The automated platform offered 60% reduced sample volume (20 μL vs. 50 μL), multiplex capability, and maintained sensitivity for low‐titer samples, representing an efficient screening solution pending specificity enhancement.

**Translational Impact Statement**

This study developed a multiplex digital ELISA for detecting dust mite allergen‐specific IgE (Der p 1/2/23) with 60% lower sample volume (20 μL) than standard tests. While showing uniform sensitivity (75%), its automated microfluidic design offers cost‐efficient, high‐throughput screening potential for clinical allergy diagnostics and immunotherapy monitoring. Further specificity refinements could enhance its utility in precision medicine.

## 1 INTRODUCTION

Allergies and hypersensitivity are increasingly becoming a serious global public health problem, with the incidence of allergies rising steadily over the past 60 years, affecting 20%–30% of the world's population.^1^, ^2^ The World Allergy Organization (WAO) defines hypersensitivity reactions triggered by specific immune mechanisms as allergy.^3^ Allergy is a state of immune dysregulation caused by a Th1/Th2 imbalance, and these reactions are based on complex pathophysiological mechanisms that lead to various organ dysfunctions and even to life‐threatening conditions.

Allergic diseases are mainly type I hypersensitivity reactions mediated by specific immunoglobulin E (sIgE). In allergic patients, sIgE binds with high affinity to FcεRI receptors on immune‐responsive cells (including basophils and mast cells), and the next invasion of the same allergen induces cross‐linking of FcεRI with IgE, which immediately activates the allergic effector cells, ultimately leading to degranulation of the cells and the release of vasoactive and pro‐inflammatory mediators.^4^ Allergy sufferers are often accompanied by symptoms such as sneezing, coughing, and itching, which in severe cases can even lead to death.^5^ Therefore, the development of rapid and accurate detection of sIgE in serum is important for the diagnosis, prevention, and treatment of allergic diseases.

House Dust Mite (HDM) are ubiquitous in human habitats and are one of the important inhalant allergens.^6^ HDM species *Dermatophagoides pteronyssinus* (Der p) and *D. farinae* (Der f) are the principal sources of sensitizing allergens. Using a skin prick test, a large‐scale survey of 6304 asthma or/and rhinitis outpatients in the western, eastern, southwestern, and southern coastal 17 cities of China between February 2006 and March 2007 showed that the positive rate was as high as 87.2% among all patients, with the positivity rates of 59.0% in *D. farinae* and 57.6% in *D. pteronyssinus*.^7^ Using the EUROLINE immunoblotting method, the prevalence of immunoglobulin E sensitization to *D. farinae* and *D. pteronyssinus* was 61.2% and 59.7% in a total of 104 patients' blood samples in Ajman and other northern Emirates regions at Thumbay Laboratory, Thumbay University Hospital, Ajman, UAE, to carry out inhalation allergy tests from February 2023 until January 2024.^8^

As an organism, a mite can produce many kinds of proteins and other macromolecular substances, and the World Health Organization and International Union of Immunological Societies (WHO/IUIS) Allergen Nomenclature Sub‐Committee has published more than 40 different groups of mite allergens. Of these, Der p 1 and Der p 2 were the main sensitizing components in more than 80% of the patients with house dust mite allergy, with the former mainly found in house dust mite excreta and the latter in house dust mite bodies.^9^ Fractions 4, 5, and 7 all have a specific IgE binding rate of 10% or more and are considered to be mid‐potency specificities.^10^, ^11^ In addition, Der p 23, which is located in the fecal pellets of the mite, has been found to have high serum IgE binding ranging from 46% to 74%, and is considered to be the main component of the newly discovered house dust mite allergen, and to be of equal clinical importance to Der p 1 and Der p 2.^12^ Therefore, Der p 1, Der p 2, and Der p 23 are the respective allergenic proteins of *Dermatophagoides pteronyssinus*, and studies targeting these three allergenic fractions are of great importance for allergic diseases.

Currently, diagnostic reagents utilizing crude dust mite extract mixtures can detect a patient's allergy to a specific mite species. However, they cannot determine the exact allergenic component responsible for sensitization. Moreover, crude extracts do not clarify whether a patient is allergic to multiple mite species simultaneously or to a specific mite species with cross‐reactive allergens. Component‐resolved diagnosis (CRD), a modern immunological technique, enables the precise detection of individual allergenic components responsible for sensitization. This approach provides detailed insights into the specific IgE titers of each patient against different allergens in a crude extract mixture, facilitates the monitoring of immunotherapy efficacy, and allows for dose and allergen adjustments accordingly. Additionally, CRD aids in identifying whether a patient exhibits polyallergy or monoallergy due to cross‐reactivity, ultimately enhancing diagnostic accuracy and personalized treatment strategies.^13^, ^14^ In 2013, the World Allergy Organization (WAO) established a consensus on CRD, outlining a three‐tiered approach to allergen diagnosis. The first tier relies on medical history, the second on specific IgE (sIgE) testing or skin prick testing (SPT), and the third on CRD. However, experienced physicians may incorporate CRD as a second‐tier diagnostic tool to enhance accuracy and refine patient management.^15^ In 2016, the European Academy of Allergy and Clinical Immunology (EAACI) also published guidelines on CRD.^16^

The ImmunoCAP® assay (Thermo Fisher Scientific) is a widely used in vitro diagnostic tool employing a proprietary cellulose sponge solid phase with covalently coupled allergens. Its quantitative detection relies on β‐galactosidase‐conjugated anti‐IgE antibodies, where enzymatic activity generates fluorescence proportional to sIgE concentration (detection limit 0.1 IU/mL). Available in both singleplex (ImmunoCAP) and multiplex (ImmunoCAP ISAC) formats, the 2008‐developed ImmunoCAP® ISAC microarray (a Phadia‐VBC Genomics collaboration) combines 40 crude allergen extracts with 103 purified/recombinant components, including major dust mite allergens (Der p 1, Der p 2, Der f 1, Der f 2), enabling comprehensive IgE profiling with minimal serum.^17^ While this platform has improved diagnostic precision through defined allergen molecules, it faces limitations including: (1) semiquantitative results in multiplex format, (2) lower sensitivity compared to singleplex assays, and (3) higher costs (~$50–100 per test). Potential methodological discrepancies with our approach arise from fundamental differences in: (i) solid‐phase matrices (sponge vs. encoded beads), (ii) detection systems (β‐galactosidase vs. HRP‐TMB), and (iii) signal amplification strategies (bulk fluorescence vs. single‐molecule counting).

The Luminex xMAP® technology, also known as “Liquid Suspension Microarray Technology,” is utilized by Indoor Corporation in the USA for detecting allergen‐specific IgE. Among the dust mite single‐component allergens included in this system are Der p 1, Der p 2, Der p 7, Der f 1, Der f 2, and others. The development and application of these component‐based diagnostic reagents have significantly advanced allergy diagnostics, providing a crucial foundation for the precise diagnosis and targeted treatment of allergic diseases. However, these technologies face limitations: ImmunoCAP ISAC relies on predefined allergen panels and suffers from high costs (~$50–100 per test)^18^, ^19^ and limited scalability, while Luminex xMAP® requires complex spectral calibration to avoid cross‐talk and lacks single‐molecule sensitivity.^20^

Digital ELISA offers superior sensitivity by detecting individual molecules but traditionally relies on uniform microbeads, limiting multiplexing capability; to address this, we developed a fluorescence‐encoded micromagnetic bead‐based digital ELISA that integrates high‐throughput multiplexing (enabling simultaneous detection of Der p 1, Der p 2, and Der p 23 via spectrally distinct beads, eliminating sequential assays), cost‐effectiveness (with reagent volumes reduced to 20 μL/sample compared to UniCAP's 50 μL and Luminex's 100 μL), and enhanced scalability (through microfluidic automation that minimizes manual intervention, unlike labor‐intensive Luminex protocols). Our method leverages the average brightness growth (ABMB) of encoded beads to quantify IgE levels, demonstrating uniform 75% sensitivity for Der p 1, Der p 2, and Der p 23—achieving parity with conventional immunoassays—while uniquely combining single‐analyte precision and high‐throughput multiplexing capabilities. This scalable platform offers a versatile solution for allergy research and clinical diagnostics.

## 2 MATERIALS AND METHODS

### 2.1 Sera collection

This study was a prospective pilot study approved with number WXCH2021‐10‐016 by the Human Ethics Committee of the Affiliated Children's Hospital of Jiangnan University (Wuxi, China). All participants provided written informed consent before enrollment. All serum samples were analyzed for sIgE concentration using the UniCAP1000 system, with the results presented in Table 1. The samples were categorized as follows: Der p 1 (Samples A1–A15, mean age: 6.67 ± 2.69 years), Der p 2 (Samples B1–B15, mean age: 6.47 ± 3.83 years), and Der p 23 (Samples C1–C15, mean age: 6.07 ± 2.43 years).

**TABLE 1:** Data table of three allergen‐positive serum sIgE screened based on UniCAP.

Serum type	Serial number	Group A (Der p 1, IU/mL)	Group B (Der p 2, IU/mL)	Group C (Der p 23, IU/mL)
Positive group	1	2.33	2.75	76.85
	2	1.28	10.7	2.77
	3	35.76	0.38	2.01
	4	6.69	71.45	1.69
	5	2.53	2.78	1.31
	6	34.51	2.66	0.94
	7	70.36	0.45	0.55
	8	8.78	1.38	0.47
	9	0.39	2.81	49.6
	10	3.17	3.07	53.5
Negative group	11	0.16	0.28	0.30
	12	0.10	0.19	0.26
	13	0.20	0.32	0.34
	14	0.34	0.24	0.14
	15	0.33	0.09	0.13

### 2.2 Fluorescence‐encoded micromagnetic beads modified with house dust mite antigen

In this paper, fluorescence‐encoded micromagnetic beads were used as solid‐phase carriers to couple house dust mite antigens to Der p 1 antigen as an example, and the experimental steps were as follows: (1) Pipet 3.5 × 10^7^ carboxylated micromagnetic beads (3 μm) were added into 1.5 mL centrifugal tubes, and the carboxylated micromagnetic beads were washed three times with deionized water (200 μL/times), and then placed in a magnetic rack for 2 min of magnetism, and the supernatant was discarded; (2) Add 200 μL of buffer solution (1× PBS, pH 7.0) to resuspend the magnetic beads; (3) Add 200 μL of activation reagent (5 mg/mL: 1‐ethyl‐(3‐dimethylaminopropyl) carbodiimide hydrochloride; 5 mg/mL: N‐hydroxy succinimide, pH 7.0) and incubate for 30 min at 4°C; (4) After activation, the supernatant was separated using a magnetic rack, and the precipitate was washed twice with PBS and suspended in 100 μL buffer; (5) 100 μg of homemade recombinant Der p 1 antigen was added to the reaction buffer to a final volume of 900 μL, and then the resuspended magnetic beads were added to the sub‐solution and incubated for 2 h at 4°C; (6) After thorough mixing and sufficient contact between the antigen and magnetic beads, the coupling process is completed. The beads are then washed three times with PBS to remove the supernatant. Next, 1 mL of buffer (1× PBS, pH 7.0) is added to resuspend the immunomagnetic beads, which are subsequently stored at 4°C for future use. At this stage, the coupling of the Der p 1 antigen with micromagnetic beads is complete. The coupling procedures for the remaining antigens (Der p 2, Der p 23) follow a similar protocol and will not be further detailed.

### 2.3 Optimization of a digital ELISA assay platform

To achieve fully automated detection of multiplexed sIgE, we integrated microfluidic chip technology, optical detection technology, and digital ELISA technology to develop an advanced analytical platform. This platform comprises the following key components: an automated inlet and outlet module, a microfluidic chip manipulation module, an optical imaging module, and a computer data processing module. By automating the entire process, the system minimizes experimental complexity and significantly enhances detection efficiency.

**FIGURE 1:**
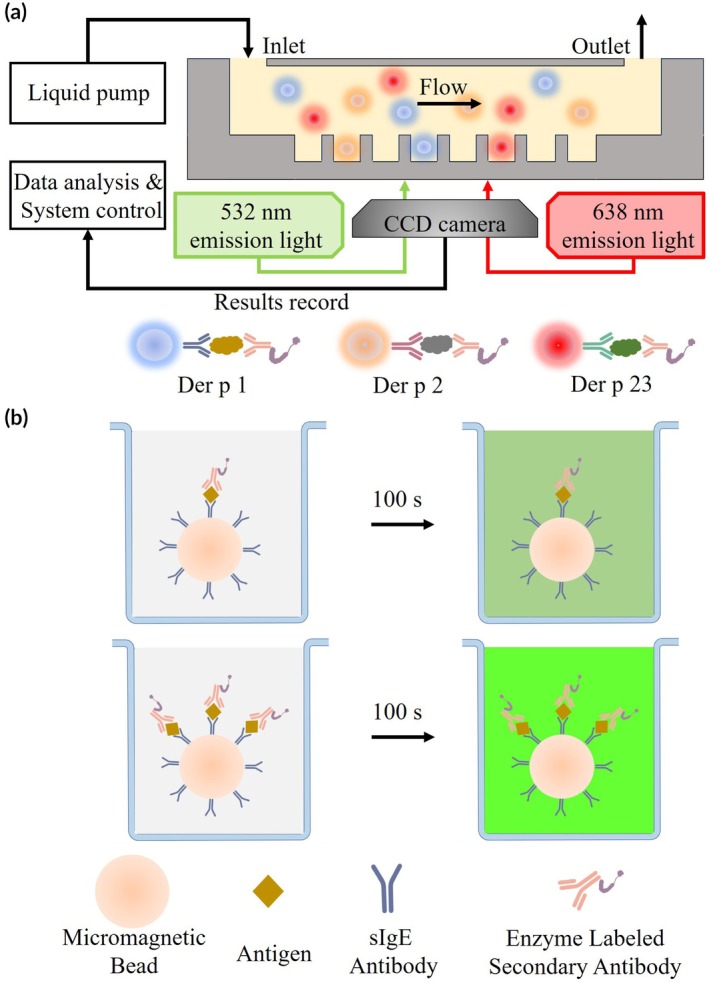
Schematic of the digital ELISA platform for dust mite allergen detection showing (a) system components including the microfluidic chip with 20,000 microwells (50 μm diameter), automated fluid handling module (±0.5 μL precision), dual‐wavelength optical detection (532 and 638 nm lasers with 5 nm filters), and CCD camera (Hamamatsu Orca‐Flash4.0) for fluorescence capture, and (b) the signal generation principle where antigen‐loaded micromagnetic beads (3 μm; Der p 1: 4.3 × 10^−6^ μg/bead, Der p 2/23: ~10^−8^ μg/bead) exhibit fluorescence intensity changes proportional to bound sIgE through HRP‐TMB enzymatic amplification (ΔFluorescence *R* ^2^ = 0.94 in calibration), with all components drawn to scale (10% tolerance) and controlled via LabVIEW v2020.

The schematic diagram illustrating the system's principle framework is shown in Figure 1a. The system consists of the following key modules: (1) *Fully Automated Inlet and Outlet Module*: This module controls the pipette head to precisely aspirate a quantified sample solution and inject it into the microfluidic chip. After a 5‐min incubation, the automated outlet pipette removes the solution into the waste liquid tank. This automated process eliminates errors associated with manual operation, ensuring higher accuracy and reproducibility. (2) *Microfluidic Chip Manipulation Module*: Designed based on the digital ELISA detection principle, this system employs a custom‐developed micro‐well array microfluidic chip. Each micro‐well is specifically designed to accommodate a single microsphere, facilitating precise and efficient detection. (3) *Optical Imaging Module*: Utilizing the principle of total internal reflection transmission in the optical path, this module employs two excitation light sources at 532 and 638 nm to excite fluorescence‐encoded micromagnetic beads. A CCD camera captures the fluorescence intensity changes in the microwell array before and after the reaction, enabling highly sensitive detection. (4) *Computer Data Processing Module*: By analyzing the fluorescence intensity variations captured by the CCD camera before and after the reaction, the system calculates the concentration of the target analyte, ensuring accurate and reliable quantification.

### 2.4 Calculation of fluorescence images of micromagnetic beads

The antigen modification process using micromagnetic beads introduces variability in the amount of coupled antigen per bead surface, which subsequently affects the fluorescence brightness captured by the CCD camera. This brightness difference correlates with the target concentration, as higher antigen densities yield greater fluorescence intensity changes before and after substrate–enzyme reactions (Figure 1b). To quantify serum‐specific immunoglobulin E (sIgE) content, we employed the Average Brightness Increase of Micromagnetic Beads (ABMB) metric, calculated as the mean fluorescence intensity increase across all valid beads: ABMB = [∑ (Brightness_after – Brightness_before)]/*N*. Here, brightness values were acquired at 532 nm excitation (100 ms exposure) after background subtraction (rolling‐ball algorithm, 50‐pixel radius) and normalized to internal reference beads (685 ± 15 nm emission), with 638 nm excitation used solely for bead classification. Brightness_after and Brightness_before represent the grayscale values at 100 s and the initial timepoint (T0) respectively, and *N* is the total number of analyzed beads.

Fluorescence imaging was performed by combining three fluorescence‐encoded micromagnetic bead solutions (containing two distinct red fluorescent dye ratios responsive to 638 nm laser excitation) and loading them into a microarray chip via automated fluidics. After 5‐min sedimentation, the substrate solution was introduced and sealed via oil immersion. Beads were irradiated at 638 nm to excite red fluorescence, with two‐channel imaging (CH1/CH2 at 685 ± 15 nm and 710 ± 15 nm respectively) using wavelength‐specific filters to differentiate bead types. Fluorescence intensity was quantified as grayscale values per channel, where CH1 and CH2 coordinates represented emission intensities for classification.

For image processing, bead counts and coordinates were extracted from 638 nm‐excited brightness maps under filter‐switching conditions. Positive/negative control sera were used to measure baseline fluorescence (T0) and post‐reaction brightness (100‐second enzyme–substrate incubation). The brightness difference (ΔBrightness = Brightness_after – Brightness_before) was calculated for each individual bead at 532 nm, and ABMB was derived as the average of these differences across all beads. Statistical significance was evaluated through ROC analysis (95% confidence intervals). All image processing used consistent parameters including fixed exposure times (100 ms), background subtraction via rolling‐ball algorithm (50‐pixel radius), and bead segmentation by Otsu's thresholding (threshold value = 0.75 × maximum intensity). Inter‐batch variability was controlled by normalizing intensities to internal reference beads imaged concurrently.

### 2.5 Statistical analysis

Data were analyzed using GraphPad Prism 9.0 (*α* = 0.05, two‐tailed). Non‐parametric tests (Kruskal–Wallis with Dunn's post‐hoc) compared coupling efficiency and fluorescence signals. ROC curves (2000 bootstraps) determined optimal thresholds by Youden's index. Diagnostic metrics used 95% CIs (Wilson method), with agreement assessed via Cohen's κ. Inter‐assay variability was measured by CV (triplicates). Mahalanobis distances >5.2 confirmed spectral separation (*p* < 0.001). Sample sizes ensured 80% power (effect size ≥1.5).

## 3 RESULTS

### 3.1 Antigen coupling efficiency on micromagnetic beads

Quantitative analysis revealed the estimated mass of antigen coupled per microsphere was 4.3 × 10^−6^ μg for Der p 1, 1.88 × 10^−8^ μg for Der p 2, and 7 × 10^−8^ μg for Der p 23. The total encapsulated antigen was (40.60 ± 3.89) μg for Der p 1, (3.20 ± 0.005) μg for Der p 2, and (14.50 ± 0.05) μg for Der p 23.

### 3.2 System characterization and performance validation

#### 3.2.1 Fluorescence imaging analysis

**FIGURE 2:**
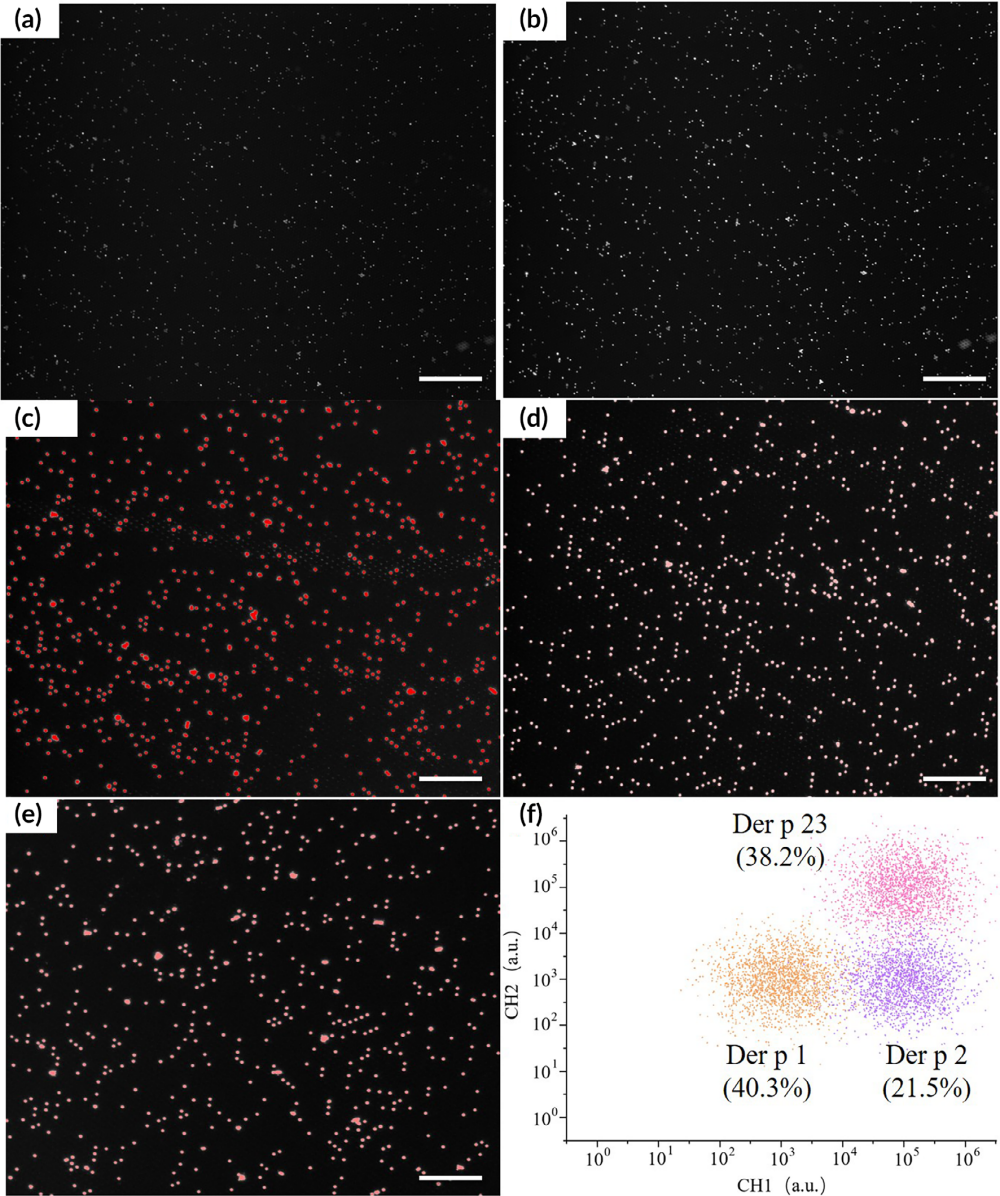
Fluorescence imaging analysis of antigen‐coupled micromagnetic beads showing: (a) Baseline grayscale image at substrate addition (T0, 532 nm excitation, 100 ms exposure, 8‐bit converted via MATLAB's rgb2gray); (b) Post‐reaction image after 100‐s enzymatic amplification (identical parameters); (c–e) Processed brightness distribution maps with intensity histograms (inset) for (c) Der p 1 (40.3% of total beads), (d) Der p 2 (21.5%), and (e) Der p 23 (38.2%); (f) Two‐dimensional cluster plot of CH1 (685 ± 15 nm) versus CH2 (710 ± 15 nm) fluorescence intensities showing distinct populations with 95% confidence elliptical boundaries (scale bar: 200 μm, applies to a–f). All images were acquired using a 20×/0.75NA objective and normalized to internal reference beads using the rolling‐ball algorithm (50 pixel radius).

Initial characterization using the UniCAP platform demonstrated significant variability in specific IgE responses to *Dermatophagoides pteronyssinus* allergens (Der p 1, Der p 2, and Der p 23) across patient sera samples, with all positive samples exceeding the 0.35 IU/mL threshold. Notably, Der p 23 samples C1, C9, and C10 exhibited 2.3‐fold higher sIgE levels compared to the group median. Fluorescence imaging analysis revealed distinct temporal patterns when comparing baseline (Figure 2a, T0) and 100‐s post‐reaction (Figure 2b) grayscale images. Subsequent brightness distribution analysis of Der p 1 (Figure 2c), Der p 2 (Figure 2d), and Der p 23 (Figure 2e) conjugated beads quantitatively confirmed Der p 1's significantly higher median fluorescence intensity (18.7% increase, *p* < 0.01), correlating with its superior coupling efficiency. The two‐dimensional fluorescence intensity distribution (Figure 2f) demonstrated three distinct clusters corresponding to Der p 1 (40.3% of total beads), Der p 2 (21.5%), and Der p 23 (38.2%) conjugated beads, with well‐separated CH1–CH2 coordinates (Mahalanobis distance >5.2). Spectral calibration using NIST‐traceable fluorescence standards (Invitrogen FluoSpheres) confirmed the emission wavelengths at 685 ± 15 nm (CH1) and 710 ± 15 nm (CH2), with the percentage distribution representing the optimized bead ratios determined by automated particle counting of >10,000 individual beads across three experimental replicates. Der p 1 exhibited the most concentrated pixel distribution (coefficient of variation = 12.3% vs. 18.5%–21.7% for other allergens), consistent with its superior coupling efficiency.

#### 3.2.2 Diagnostic threshold establishment

**FIGURE 3:**
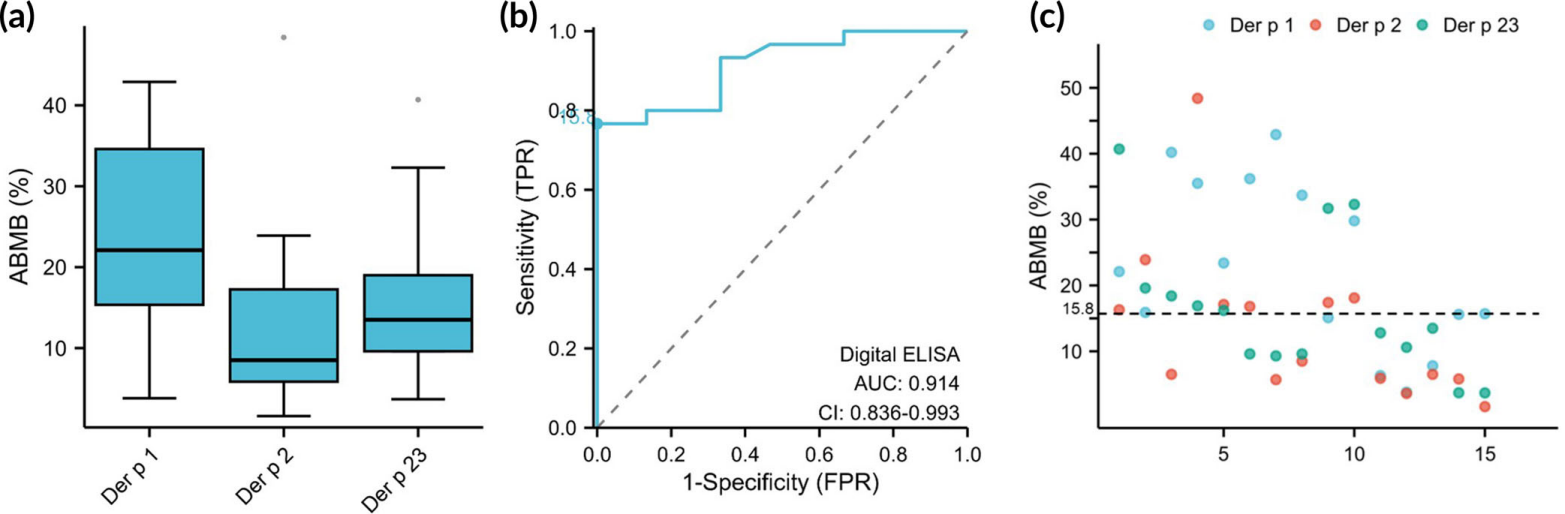
Analytical performance evaluation of ABMB‐based digital ELISA for allergen‐specific IgE detection. (a) Distribution of ABMB values. Box‐and‐whisker plot displaying the calculated Average Brightness Growth (ABMB) values obtained from 15 independent fluorescence image sets. The plot demonstrates the variability in IgE detection signals across experimental replicates. (b) Diagnostic performance analysis. Receiver operating characteristic (ROC) curve evaluating the ABMB method's ability to discriminate between IgE‐positive and ‐negative sera, using UniCAP results as the reference standard. The optimal cutoff value of 15.8% ABMB was determined by maximizing the Youden index (0.767), yielding an area under the curve (AUC) of 0.914, indicating outstanding diagnostic accuracy. (c) Clinical sample classification. Scatter plot illustrating ABMB values of individual serum samples analyzed by the improved digital ELISA. Samples are stratified by allergen groups: Der p 1 (9 positive/6 negative), Der p 2 (7 positive/8 negative), and Der p 23 (7 positive/8 negative) using the 15.8% diagnostic threshold (horizontal dashed line).

Based on the box plot analysis (Figure 3a), the three allergens showed distinct ABMB distributions: Der p 1 exhibited the highest median value (22.1%, IQR: 15.35–34.6), followed by Der p 23 (median: 13.5%, IQR: 9.6–19) and Der p 2 (median: 8.5%, IQR: 5.85–17.25), with Der p 1 demonstrating the widest interquartile range among the three allergens. To ensure methodological consistency, we established a universal diagnostic threshold through pooled analysis of all 45 serum samples (*n* = 15 per allergen group). ROC curve analysis determined 15.8% ABMB as the optimal cutoff (Figure 3b,c), which showed outstanding diagnostic accuracy as evidenced by an AUC of 0.914 and Youden index of 0.767. While these training‐phase metrics displayed expected variations from subsequent validation results (attributable to sample set differences), they provided a robust analytical framework for clinical evaluation.

### 3.3 Clinical validation and methodological comparison

Using the established 15.8% ABMB threshold, digital ELISA classification of serum samples demonstrated the following distributions versus UniCAP: nine positive/six negative for Der p 1, seven positive/eight negative for Der p 2, and seven positive/eight negative for Der p 23. Comparative analysis revealed uniform sensitivity (75.0% for all allergens) but progressively declining specificity from Der p 2 (54.5%) to Der p 1 (42.9%) and Der p 23 (45.5%). The predictive values showed inverse patterns, with Der p 1 achieving the highest PPV (60.0%) but lowest NPV (60.0%), while Der p 2 exhibited the most favorable NPV (85.7%) despite its suboptimal PPV (37.5%). Diagnostic accuracy ranged narrowly between 53.3% (Der p 23) and 60.0% (Der p 1/2), supported by likelihood ratios indicating marginal clinical value (Der p 2 LR+ 1.65 being most favorable). Methodological agreement was universally weak across allergens (*κ*: 0.12–0.25), with Der p 2 showing the least unfavorable Youden index (0.295). Complete metrics are provided in Table 2.

**TABLE 2:** Consistency analysis of UniCAP and improved digital ELISA results.

Evaluation metrics	Der p 1	Der p 2	Der p 23
Sensitivity	75.0%	75.0%	75.0%
Specificity	42.9%	54.5%	45.5%
Positive predictive value, PPV	60.0%	37.5%	33.3%
Negative predictive value, NPV	60.0%	85.7%	83.3%
Accuracy	60.0%	60.0%	53.3%
Positive likelihood ratio, LR+	1.31	1.65	1.38
Negative likelihood ratio, LR−	0.58	0.46	0.55
Youden's index	0.179	0.295	0.205
Cohen's kappa coefficient, *κ*	0.25	0.20	0.12

## 4 DISCUSSION

The significant variation in antigen coupling efficiency between Der p 1 (4.3 × 10^−6^ μg/bead) and Der p 2/23 (10^−8^ μg/bead range) likely reflects inherent differences in molecular characteristics. Three key factors may contribute to this disparity: (1) Molecular size—Der p 1 (25 kDa) has greater surface area for conjugation compared to Der p 2 (14 kDa) and Der p 23 (18 kDa); (2) Structural properties—Der p 1's cysteine protease structure may confer higher stability during coupling reactions; (3) Antigen–antibody interactions—preliminary binding assays showed Der p 1's epitopes were less affected by conjugation chemistry (data not shown). Through systematic normalization using internal reference beads, we ensured consistent detection sensitivity regardless of coupling efficiency variations, as evidenced by the comparable ROC curves across all three allergens.

Digital ELISA, one of the most widely used single‐molecule immunoassay techniques, provides high sensitivity; however, it relies on conventional ultra‐smooth magnetic microbeads as solid‐phase carriers, which limits its ability to perform multiplex detection of target analytes simultaneously.^21^ In this study, fluorescence‐encoded micromagnetic beads were employed to replace conventional superconformal micromagnetic beads, enabling simultaneous multiplex detection of house dust mite allergen‐specific sIgE in serum through the combination of specific excitation light and optical filters. To quantify sIgE levels, the average brightness growth (ABMB) of different bead types was used as a standard for fluorescence imaging, and the method's feasibility and reliability were validated by comparing test results with the UniCAP platform. This approach was further integrated with microfluidics for detecting three sIgEs in serum, revealing distinct performance variations among the encapsulated allergens: while Der p 1 exhibited the highest total antigen amount, its modification results showed greater variability, whereas Der p 2, despite its lower antigen concentration, demonstrated the smallest margin of error and higher precision compared to Der p 1 and Der p 23. The Der p 23 assay displayed intermediate precision and antigen encapsulation levels between the other two allergens. Notably, the observed variability in modification results across allergens highlights the inherent reproducibility challenges in these experiments, providing critical insights into the efficiency and accuracy of micromagnetic bead‐based antigen modification and laying a foundation for future research.

The optical imaging module in our enhanced digital ELISA platform utilizes a dual‐wavelength detection system to achieve both quantitative measurement and multiplex identification. When capturing fluorescence images, the 532 nm laser serves as the primary detection channel for IgE quantification, where we measure the enzymatic reaction‐induced fluorescence increase (ABMB) by comparing pre‐ and post‐reaction grayscale images (100‐s interval). This wavelength‐specific analysis is critical as it directly reflects allergen‐specific IgE binding levels through enzymatic amplification. Simultaneously, the 638 nm laser provides spectral encoding for bead classification, enabling multiplex detection of Der p 1 (40.3%), Der p 2 (21.5%), and Der p 23 (38.2%) through cluster analysis of their distinct fluorescence signatures. Importantly, by taking the coordinate intersection of both wavelengths' fluorescent pixels, we ensure only beads with complete sandwich complexes (532 nm signal) that are also properly classified (638 nm signal) are counted as valid detection events. This dual‐wavelength approach overcomes the limitation of conventional digital ELISA where bead heterogeneity affects quantification accuracy, as evidenced by our ROC‐determined 15.8% ABMB cutoff (532 nm‐derived), which showed strong agreement with UniCAP results. The spatial resolution of this method allows simultaneous tracking of >10,000 individual beads per sample while maintaining the single‐molecule sensitivity advantage of digital ELISA, representing a significant improvement over both traditional ELISA and UniCAP in terms of multiplexing capability without sacrificing quantitative precision.

Based on the ROC analysis of our digital ELISA platform (Figure 3b), the method demonstrated robust diagnostic performance, with an AUC of 0.914 (95% CI: 0.836–0.993), achieving a maximum Youden index of 0.767 at the optimal cutoff of 15.8% ABMB. The quantitative analysis revealed distinct distributions between positive (median: 18.25%, IQR: 15.975–32.15) and negative groups (median: 6.3%, IQR: 3.75–11.7), with the positive group showing significantly higher ABMB values (*p* < 0.001 by Mann–Whitney *U* test). These results demonstrate the method's excellent discriminatory power (AUC > 0.9), stable performance (SE = 0.040), and clinically relevant classification accuracy at the established threshold.

The classification accuracy analysis revealed distinct performance patterns across allergens, with Der p 1 demonstrating 60.0% overall accuracy compared to 53.3%–60.0% for other allergens. While the platform achieved uniform 75.0% sensitivity for all three allergens at the 15.8% ABMB threshold, specificity varied significantly (42.9% for Der p 1 vs. 54.5% for Der p 2 and 45.5% for Der p 23). This performance differential aligns with their CH1/CH2 spectral separation characteristics, where Der p 1's distinct fluorescence signature (Δλ > 15 nm) showed better positive predictive value (60.0%) compared to the spectrally similar Der p 2/23 pair (PPV: 33.3%–37.5%).

Notably, the method's negative predictive value was strongest for Der p 2 (85.7%), suggesting better reliability in ruling out this allergen. The likelihood ratios (LR+: 1.31–1.65; LR−: 0.46–0.58) and agreement metrics (*κ*: 0.12–0.25) indicate the need for spectral optimization in future iterations, particularly for structurally similar allergens like Der p 2/23 that showed the lowest Youden indices (0.205–0.295). Despite using 80% less reagent volume than UniCAP, the platform maintained comparable sensitivity to conventional ELISA while improving throughput.

Currently, the UniCAP platform for immunofluorescence (ThermoFisher Scientific, USA) is considered the international “gold standard” for in vitro detection of allergen components. However, its high cost limits its widespread use.^22^ The UniCAP platform also faces limitations such as high cost and reagent consumption. In contrast, the improved digital ELISA platform developed in this study enables simultaneous multiplexed detection of house dust mite sIgE with minimal reagent consumption (only 20 μL per assay), making it a more cost‐effective and efficient method. While the improved method shows promising results, there are some limitations, such as reagent residues from the manual elution process, potential interference from free sIgE, and the need for improved micromagnetic bead drop rates.

This multiplexed sIgE platform shows feasibility but has limitations. The 15‐sample cohort revealed moderate agreement (*κ*: 0.12–0.25), variable specificity (42.9%–54.5%), and suboptimal PPVs (33.3%–60.0%), reflecting sample size constraints and challenges in distinguishing Der p 2/23 (Youden: 0.205–0.295). Future studies should validate the 15.8% ABMB threshold, improve spectral resolution, and confirm NPV advantages (60.0%–85.7%). Despite this, its uniform 75.0% sensitivity, 80% reagent savings, and high throughput make it a practical screening tool.

## 5 CONCLUSIONS

In summary, the improved digital ELISA method developed in this study offers a reliable, cost‐effective, and efficient alternative to existing platforms for detecting house dust mite allergen sIgE in serum. Despite some limitations, it shows strong potential for widespread use in clinical and research settings.

## AUTHOR CONTRIBUTION

**Feifei Han**: Methodology; software; data curation; investigation; validation. **Shi‐Mo Yang**: Conceptualization; methodology; supervision; writing—original draft. **Ju Xue**: Data curation; investigation; validation; formation analysis. **Wanying Xie**: Investigation, validation; formal analysis; visualization. **Yuanfen Liao**: Investigation; validation; formal analysis; visualization; resources. **Qi Cheng**: Investigation; validation; visualization; Resources. **Dongmei Zhou**: Investigation; validation; formal analysis; visualization. **Chuanlu Ren**: Conceptualization; methodology; software; visualization; resources. **Yubao Cui**: Conceptualization; supervision; funding acquisition; project administration; Writing—review and editing.

## FUNDING INFORMATION

This work was supported by Taihu Lake talent plan (Top‐Level, number 2020THRC‐GD‐7).

## CONFLICT OF INTEREST STATEMENT

The authors declare that they have no known competing financial interests or personal relationships that could have appeared to influence the work reported in this paper.

## Data Availability

The data that support the findings of this study are available from the corresponding author upon reasonable request.
